# Mid- and long-term clinical results of surgical therapy in unicameral bone cysts

**DOI:** 10.1186/1471-2474-12-281

**Published:** 2011-12-13

**Authors:** Sébastien Hagmann, Florian Eichhorn, Babak Moradi, Tobias Gotterbarm, Thomas Dreher, Burkhard Lehner, Felix Zeifang

**Affiliations:** 1 Department of Orthopedic Surgery and Traumatology, University Hospital of Heidelberg, Schlierbacher Landstrasse 200a, 69115 Heidelberg, Germany; 2 Thoraxklinik, University of Heidelberg, Heidelberg, Germany

**Keywords:** Solitary bone cyst, Unicameral bone cyst, Simple bone cyst, Juvenile bone cyst, Curettage, Bone graft, Steroid injection

## Abstract

**Background:**

Unicameral (or simple) bone cysts (UBC) are benign tumours most often located in long bones of children and adolescents. Pathological fractures are common, and due to high recurrence rates, these lesions remain a challenge to treat. Numerous surgical procedures have been proposed, but there is no general consensus of the ideal treatment. The aim of this investigation therefore was to study the long-term outcome after surgical treatment in UBC.

**Methods:**

A retrospective analysis of 46 patients surgically treated for UBC was performed for short and mid-term outcome. Clinical and radiological outcome parameters were studied according to a modified Neer classification system. Long-term clinical information was retrieved via a questionnaire at a minimum follow-up of 10 years after surgery.

**Results:**

Forty-six patients (17 female, 29 male) with a mean age of 10.0 ± 4.8 years and with histopathologically confirmed diagnosis of UBC were included. Pathological fractures were observed in 21 cases (46%). All patients underwent surgery for UBC (35 patients underwent curettage and bone grafting as a primary therapy, 4 curettage alone, 3 received corticoid instillation and 4 decompression by cannulated screws). Overall recurrence rate after the first surgical treatment was 39% (18/46), second (17.4% of all patients) and third recurrence (4.3%) were frequently observed and were addressed by revision surgery. Recurrence was significantly higher in young and in male patients as well as in active cysts. After a mean of 52 months, 40 out of 46 cysts were considered healed. Prognosis was significantly better when recurrence was observed later than 30 months after therapy. After a mean follow-up of 15.5 ± 6.2 years, 40 patients acknowledged clinically excellent results, while five reported mild and casual pain. Only one patient reported a mild limitation of range of motion.

**Conclusions:**

Our results suggest satisfactory overall long-term outcome for the surgical treatment of UBC, although short-and mid-term observation show a considerable rate of recurrence independent of the surgical technique.

## Introduction

Simple bone cysts (SBC) or unicameral bone cysts (UBC) are benign, fluid-filled cavities most often located in the metaphyses of long bones in children and adolescents, predominantly in the proximal humerus and femur. The absolute incidence is unknown, but the rate among all bone lesions is about 3%, with a male to female ratio of 2:1 [1,2]. Most UBC are detected between the age of nine and fifteen [1,3]. Despite their first recognition by Virchow in 1876 [4], the etiology of UBC remains unknown.

There have been several theories on the pathogenesis of these lesions, ranging from an early conviction of inflammation and dysplastic processes as the main cause for UBC development [5], towards theories that propagate traumatic causes [6,7]. Others theorize that an increase in intracavitary osseous pressure accounts for cystic formation [8-10]. This elevation of intracavitary pressure may be the cause of high levels of PGE-2, IL-1β and several enzymes detected in UBC fluid [11,12]. These soluble factors may be the trigger for increased osteoblastic activity and resulting osteolysis [13,14].

The plain radiograph aspects of a lesion concentrically located in the medullary cavity of the metaphysis of a long bone with expansion in all directions [15] is virtually diagnostic of UBC and may be facilitated by the so-called fallen leaf-sign, representing a fragment of cortex in the cyst cavity, if present [16,17]. Further diagnostic measures, such as MRI, CT scan or Tc^99 ^bone scan help to differentiate these lesions from other bone tumours, especially when cysts are not located in long bones [18].

With skeletal maturity, asymptomatic UBC may spontaneously resolve [19]. However, when they are not discovered incidentally, UBC may often be revealed by pathologic fractures due to cortical destabilization. Healing of bone cysts after pathological fracture occurs rarely [20], and immobilisation and observation may lead to recurrent fractures in 62 to 82% [2,8,9]. Therefore, in most cases, surgical treatment is essential for therapeutic success.

Possible options for surgery include percutaneous decompression with cannulated screws or pins [21,22], percutaneous aspiration with injection of corticosteroids or injection of bone marrow [23-26], curettage with or without bone grafting, intramedullary nailing [27] or combinations of the above [28,29].

Attempts have been made to define factors that might promote recurrence. Young age and male sex may be associated with higher recurrence rates [30-32]. Active cysts, defined as being in direct contact to the adjacent growth plate [33], are also reported to have a higher recurrence rate than inactive (or latent) ones [6].

Due to their high recurrence rates and despite the many therapies that have been proposed, UBC remain a challenge to treat. However, clinical experience suggests that only a few adults present to orthopaedic surgeons with symptoms after UBC therapy.

The aim of this study was therefore to determine the mid-term radiographic healing after surgical treatment of unicameral bone cysts. Furthermore, we wanted to provide long-term clinical information on a collective treated in our institution for UBC.

## Material and methods

### Patients

We performed a retrospective analysis of patients treated for UBC in our institution between 1983 and 1996 via a tumour database. Patients treated after 1996 were excluded from the study to obtain a minimum follow-up of 10 years. In 2007, patients included in the analysis were systematically interviewed for clinical outcome (see below). Retrieval of patient information was approved by the Ethics committee of the medical faculty at the University of Heidelberg, Germany by the number 278/99. All patients approved the application of their data to this study. The research reported in the paper was undertaken in compliance with the Helsinki Declaration.

### Inclusion criteria

Inclusion criteria were: 1) A unicameral bone cyst diagnosed by radiological, histological and operative features. 2) At least one surgical treatment in our institution with one of the procedures listed below. 3) Retrieval of long-term clinical information in the year 2007.

### Exclusion criteria

Sixty-two patients met inclusion criteria one and two. Out of these 62 patients retrieved from our archive, 11 patients with missing information regarding long-term outcome (patients lost to follow-up, not meeting inclusion criteria three) or with missing radiographs providing mid-term outcome information (n = 5) were excluded.

### Surgery

All procedures were performed under general anaesthesia. Surgical procedures were the following: a) simple curettage, b) curettage followed by bone grafting with autograft or allograft, c) aspiration and injection with methylprednisolone, d) decompression by hollow screws. One patient was initially treated conservatively. Due to persistence of symptoms, he was later treated with curettage followed by bone grafting.

### Curettage

Access to the cavity was achieved by applying four to six drill holes to the bone just over the cystic cavity and removing a bony window with the help of a chisel. The whole of the cavity was then addressed with a bone curette and the cyst membrane was removed under x-ray control, assuring that curettage was clearly performed beyond the radiographic circumference of the cyst. The bone window was not put back into place.

### Curettage and bone graft

The technique of curettage was performed as mentioned above. Bone grafting was either autologous (iliac crest) or allogenic (acellular bone provided from the bone bank in our institution).

### Steroid injection

Steroid injection was performed in a modified technique first described by Scaglietti et al. in 1979 [34]. A single K-wire was introduced in the cavity of the cyst under x-ray control. A needle was then introduced. Emptying of cyst fluid was achieved by aspiration. An individual volume of methylprednisolone was then injected into the cavity, with the final dose adapted to the size of the cyst (average dose of 90.0 mg, SD = 68.4 mg, ranging from 10 mg to 250 mg), filling up the cavity until some fluid escaped from the bone.

### Decompression by cannulated screws

Decompression was achieved by applying up to four cannulated screws reaching the cystic cavity. The screws were inserted over x-ray controlled k-wires.

### Postoperative treatment and radiological control

Postoperative treatment varied upon localisation of the cysts, age of the patient, presence of pathologic fracture etc. and ranged from simple dressings and splints to plaster cast therapy in the more severe cases.

Radiographs were taken 1, 3, 6 and 12 months after surgery. Depending on success or recurrence, radiological evaluation was then individually modified.

### Radiographic analysis

**Figure 1:**
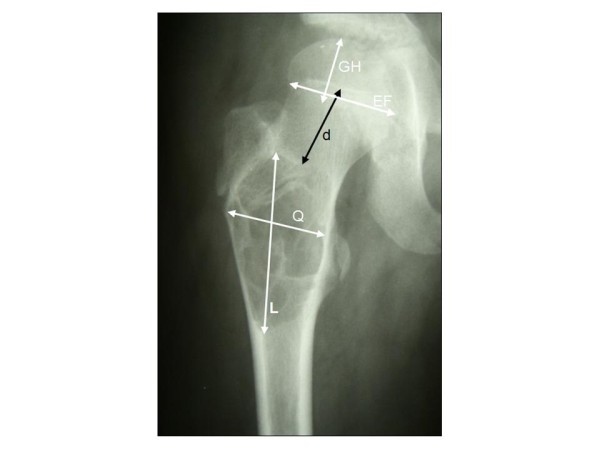
**Radiographic evaluation of UBC morphology**. The radiographs were evaluated according to the following parameters: length (L), width (Q), width of the growth plate (EF), distance from the adjacent growth plate (d) and distance from the growth plate to the joint (GH).

All radiographs taken in the course of treatment were evaluated by two blinded investigators according to the following parameters: length (L), width (Q), and depth (T, taken from a second projection) (Figure 1). By multiplication of these 3 parameters, a fourth parameter was obtained (V, being a dimensionless approximate "volume" parameter). To rule out errors caused by different x-ray magnifications, scaling of these parameters was achieved by relating all parameters to a quotient taken from the distance of the cyst to the adjacent growth plate to the width of this growth plate (EF, Figure 1). Growth and recurrence were analysed by comparing these parameters in the course of treatment.

Activity of the cyst was classified into latent and active according to Jaffe and Lichtenstein [6]. Active cysts were considered having a distance to the adjacent growth plate (d, Figure 1) of less than 10 mm, while latent cysts were considered having a distance of 10 mm or more. Postoperative radiographs were classified for successful treatment using a modification of a classification system first described by Neer [35] and modified by Chang et al. [36] (Table 1).

**Table 1:** Modified Neer classification system for postoperative radiological evaluation of unicameral bone cysts according to Chang et al

Healed	Cyst filled by formation of new bone with or without small static, radiolucent area(s) less than 1 cm in size.
**Healing with defect**	Static, radiolucent area(s) less than 50% of the diameter of the bone with enough cortical thickness to prevent fracture.

**Persistent cyst**	Radiolucent areas greater than 50% of diameter of the bone and with a thin cortical rim. No increase in cyst size.

**Recurrent cyst**	Cyst reappeared in a previously obliterated area or a residual radiolucent area has increased in size.

### Long-term clinical outcome

In the year 2007, with a minimum follow-up of 10 years, all patients were polled with a questionnaire for presence or absence of pain, activity, contentment with treatment outcome (grades 1 to 6) and necessity of further treatment outside our institution for UBC.

### Statistical analysis

Analysis was conducted using SPSS software (version 15, SPSS Inc., Chicago, IL, USA). For the analysis of dichotomous variables between two groups, a chi-square cross tab test was performed. Values of *p *< 0.05 were considered significant.

## Results

Forty-six patients (17 female, 29 male) with a mean age of 10.0 ± 4.8 years were included. An overview of patient's features and response to treatment is given in Table 2. In 42 of 46 cases, diagnosis was confirmed by positive histology in the course of first surgery. In the four remaining cases, no material for histopathological analysis had been obtained during primary therapy for technical reasons. Diagnosis was in these cases at first confirmed by typical radiographic morphology and operative observation. In these four cases, histopathological diagnosis was later confirmed in the course of revision surgery.

**Table 2:** Overview of patient's features and response to initial treatment

Patient	Sex	Age	Cyst localization	Side	Fracture	Initial treatment	Bone graft	Recurrence
AS	f	8	proximal fibula	Left	no	Curettage + bone graft	allogeneic	no

BH	m	5	distal humerus	Left	yes	conservative	none	yes

BJ	m	11	proximal femur	Left	no	curettage	none	no

BK	f	6	distal humerus	Left	yes	Curettage + bone graft	allogeneic	yes

BL	f	13	distal humerus	Left	no	Curettage + bone graft	allogeneic	no

BM	f	4	proximal femur	Left	yes	Curettage + bone graft	allogeneic	no

BS	f	5	proximal femur	Left	no	Curettage + bone graft	allogeneic	no

DA	m	4	distal fibula	Left	no	Curettage + bone graft	allogeneic	yes

EB	m	7	distal fibula	Left	yes	Curettage + bone graft	allogeneic	yes

ES	f	11	calcaneus	Right	no	curettage	none	no

ET	m	14	proximal femur	Left	no	Curettage + bone graft	allogeneic	yes

ET	m	9	calcaneus	Right	no	Curettage + bone graft	allogeneic	no

GA	m	9	distal humerus	Right	no	Curettage + bone graft	allogeneic	yes

GE	f	19	medial humerus	Right	no	Curettage + bone graft	autologous	no

GM	m	6	distal humerus	Right	yes	Curettage + bone graft	allogeneic	yes

GS	f	2	proximal femur	Left	no	Curettage + bone graft	allogeneic	yes

HE	f	14	distal tibia	Right	yes	Curettage + bone graft	autologous	no

HF	m	11	proximal femur	Right	no	Curettage + bone graft	allogeneic	no

HH	f	22	proximal femur	Left	no	Curettage + bone graft	autologous	no

HM	m	8	distal humerus	Left	yes	Curettage + bone graft	allogeneic	no

HSa	m	13	distal humerus	Right	yes	Curettage + bone graft	allogeneic	no

HSb	m	13	distal humerus	Right	yes	Curettage + bone graft	autologous	yes

JA	m	14	distal humerus	Left	yes	curettage	none	no

JW	m	12	proximal femur	Left	yes	Curettage + bone graft	autologous	no

KA	m	7	distal humerus	Left	yes	Curettage + bone graft	allogeneic	yes

KC	m	10	proximal femur	Right	no	Curettage + bone graft	allogeneic	no

KD	f	12	proximal fibula	Left	yes	cortisone instillation	none	no

KF	m	10	distal humerus	Right	yes	cortisone instillation	none	yes

KJ	m	23	proximal femur	Left	no	Curettage + bone graft	allogeneic	no

KO	m	6	proximal femur	Right	no	Curettage + bone graft	allogeneic	no

LF	m	5	distal fibula	Left	yes	Curettage + bone graft	autologous	yes

LM	f	6	distal fibula	Left	yes	cortisone instillation	none	yes

MC	m	10	proximal tibia	Left	no	Curettage + bone graft	allogeneic	no

NU	f	19	ilium	Left	no	Curettage + bone graft	allogeneic	no

RC	f	13	calcaneus	Left	no	Curettage + bone graft	autologous	no

RMa	m	8	proximal femur	Right	yes	Curettage + bone graft	allogeneic	no

RMb	m	9	distal humerus	Left	no	Curettage + bone graft	allogeneic	yes

SC	m	11	metacarpal	Right	no	Curettage + bone graft	allogeneic	yes

SS	m	10	distal humerus	Left	no	screws	none	no

VF	f	13	proximal radius	Right	no	Curettage + bone graft	allogeneic	no

VK	m	16	calcaneus	Left	no	Curettage + bone graft	autologous	no

VT	m	12	proximal femur	Left	no	Curettage + bone graft	autologous	yes

WC	f	4	distal humerus	Right	no	screws	none	yes

WM	m	6	distal humerus	Right	yes	screws	none	yes

WS	m	10	distal humerus	Left	yes	screws	none	no

ZM	f	2	distal tibia	Left	no	curettage	none	no

The cysts were mainly located in long bones (40/46), predominantly in the femur (n = 17) and the humerus (n = 16). The other localisations among long bones were the tibia (n = 3), fibula (n = 3) and radius (n = 1). In the long bones, cysts were predominantly located in the proximal parts (n = 32). Four cysts were located in the calcaneus, one in the metacarpal and one in the ilium. Twenty-nine of the 46 cysts were located on the left side of the body, while only 17 were located on the right side.

Thirty-eight patients (83%) presented to our institution because of pain. Out of the total number of 46 patients, 21 were found to have a pathological fracture (46%). 17 patients experienced pain without fracture. In the remaining 8 patients presenting to our institution, UBC had been discovered incidentally. These patients had not encountered any symptoms in the past. In 23 cases, medical consultation took place within the first weeks of the onset of pain. Out of these, 15 pathological fractures could be detected. Medical consultation was significantly faster in case of a pathological fracture (*p *< 0.01). In the remaining 23 patients, mean duration until medical consultation was 16.3 ± 20.2 weeks.

**Figure 2:**
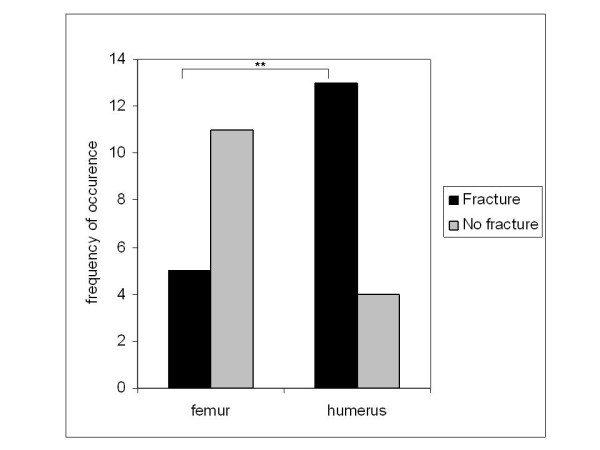
**Frequency of pathologic fractures related to localization of UBC**. Fractures were significantly more often observed in patients with UBC located in the humerus (*p *= 0.009).

Pathological fractures were significantly more often detected in the humerus than in the femur (13/17 vs. 5/16, *p *= 0.009) (Figure 2). No significant difference was observed between pathological fractures in male and female patients (15/29 vs. 6/17, *p *= 0.28).

**Figure 3:**
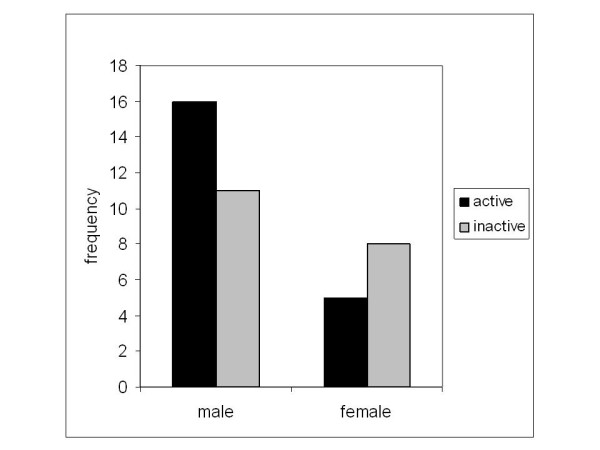
**Frequency of active cysts related to male and female sex**.

Upon evaluation of the initial x-rays taken before the beginning of the treatment, 21 cysts were classified as active (distance to the adjacent growth plate less than 10 mm), while 19 cysts were classified as inactive (or latent). In the remaining six cases (4 female patients, 2 male patients), due to the localization of the cysts, classification into active or inactive was not feasible. Active cysts were not significantly more often found in male than in female patients (16 out of 27 classified cysts vs. 5 out of 13 classified cysts, *p *= 0.217, Figure 3).

**Figure 4:**
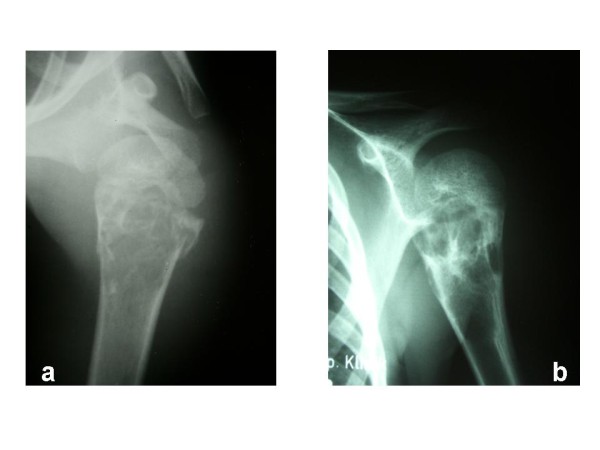
**(**a**) Pathological fracture of the proximal humerus in a male patient (5 y) initially treated conservatively**. (**b**) 5 months later, the fracture is consolidated. However, the cyst shows significant growth progression.

All patients received at least one surgical treatment in our institution. One patient was initially treated conservatively for a pathological fracture of the proximal humerus. Fracture consolidation was attained, but the patient remained symptomatic with pain. Radiographs showed a growing cyst (Figure 4a and 4b), therefore, curettage and bone grafting were performed. In total, as a primary surgical therapy, curettage and bone grafting were performed in 35 cases. 9 patients were treated with autologous bone graft from the iliac crest, in the 26 remaining cases, bone grafting was performed with acellular bone derived from our bone bank.

In four cases, curettage was performed without bone grafting. Another three patients were treated by corticoid injections, while in four patients, cannulated screws were applied.

Recurrent fractures were observed in nine cases (three femur, six humerus), with no significant difference among the different procedures.

Overall recurrence rate after primary surgical therapy was 39% (18/46) for all surgical procedures, recurrence being defined as requiring further surgical treatment. In eight of these patients (17.4%), a second recurrence was observed, while in two cases (4.3%), three repetitive surgeries had to be carried out. These cases were treated by curettage and bone grafting.

Recurrence rate for curettage and bone grafting was 37% (13/35). There was no significant difference in outcome between the group treated with autologous (n = 26) bone graft and allograft (n = 9). The recurrence rate for curettage alone was 0% (4/4 cysts considered "healed" in follow-up). These cysts had been located in the distal tibia, the distal fibula, the distal humerus and the proximal femur. Two out of 3 cysts showed recurrence after corticoid instillation. Two out of 4 cysts showed recurrence after decompression with cannulated screws.

**Figure 5:**
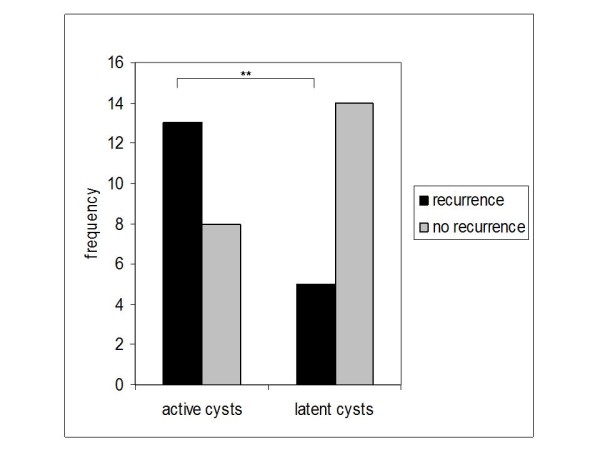
**Frequency of recurrence related to preoperative classification into active and latent**. Recurrence was significantly higher in patients with UBC classified as active (*p *= 0.025).

Independent from treatment, active cysts showed a significantly higher recurrence rate than latent ones (13/21 vs. 5/19, *p *= 0.025, odds ratio 2.5, Figure 5). In patients presenting with pathologic fractures, probability of recurrence was significantly elevated (*p *= 0.024, odds ratio 4.66). Patients who were younger than 10 years when diagnosed with UBC had a significantly elevated probability of recurrence (*p *= 0.004, odds ratio 6.52). Interestingly, when recurrence occurred later than 30 weeks after the last surgery, prognosis was significantly better compared to recurrence within 30 weeks after surgery: a second recurrence was only observed in one out of 14 patients of the former compared to 9 out of 12 in the latter (*p *< 0.01, odds ratio 40). Recurrence was higher in male patients (14/29, 48%) than in female patients (4/17, 28%), but this was not statistically significant (*p *= 0.09).

**Figure 6:**
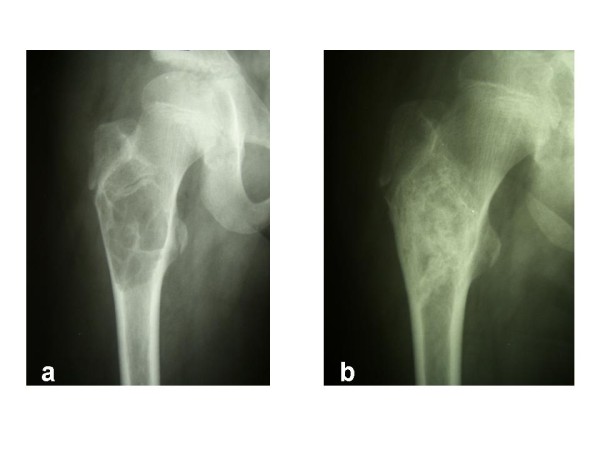
**(**a**) 12-year-old male patient treated by curettage and bone grafting for UBC of the right proximal femur**. (**b**) 7 month later, UBC was considered "healed" with complete consolidation of the graft.

**Figure 7:**
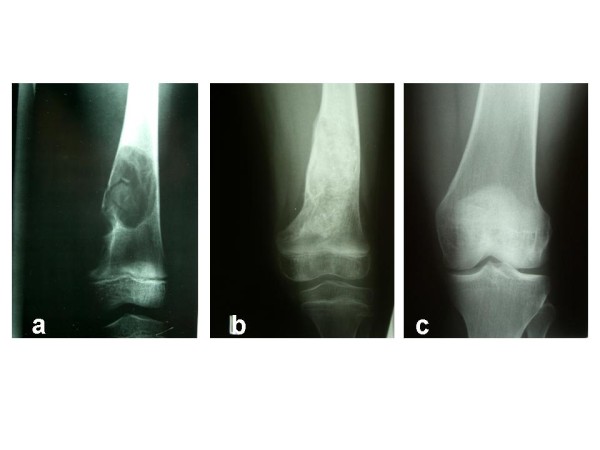
**(**a**) 6-year-old female patient initially treated by injection of methylprednisolone**. The picture shows the situation before the first injection. (**b**) 7 months later (3 month after revision curettage and bone grafting for pathological fracture of the distal femur in the course of corticoid therapy). (**c**) 16 years after the initial radiograph. No aspect of UBC is remaining.

**Figure 8:**
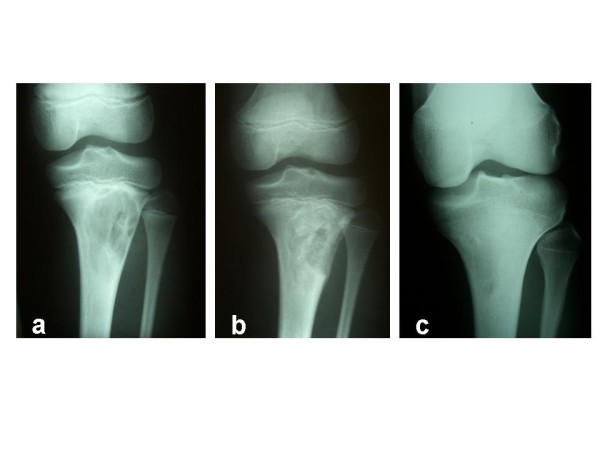
**11-year-old male patient initially treated by curettage and homologous bone grafting**. (**a**) Before treatment. (**b**) 2 months later after surgery. (**c**) 8 years after surgery. Some aspect of UBC is remaining, while the patient is asymptomatic.

**Figure 9:**
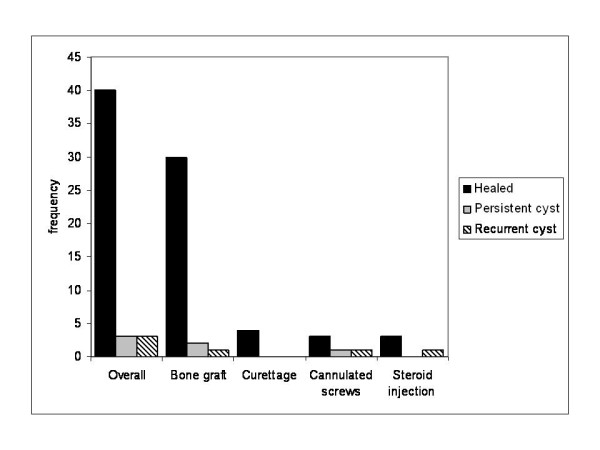
**Midterm outcome after 52 months for the different surgical therapies**.

To evaluate radiographic mid-term results, the last radiographs taken in the course of treatment were analysed. After a mean clinical and radiological follow-up of 52 ± 48.6 months, 40 out of 46 patients were classified "healed" according to the classification system mentioned above. Three patients showed "persistent cysts", while three patients showed "recurrent cysts", but declined further therapy. Three cases are demonstrated in Figures 6, 7, 8. Figure 9 outlines overall and specific treatment outcome. No significant difference was observed between the different therapies.

After a mean follow-up of 15.5 ± 6.2 years, all 46 patients were polled on long-term clinical outcome. Forty patients reported to have no pain and no functional limitations, five reported mild and casual pain, and one patient reported a mild limitation of range of motion in the shoulder joint without pain. In these latter six patients, medical consultations had not been able to reveal the agent of pain, notably, no recurrence of UBC had been detected. The six symptomatic patients were all male and had been treated with curettage and bone grafting. The localizations of the cysts in these patients were: proximal humerus (n = 2), proximal femur (n = 2), distal tibia (n = 1) and calcaneus (n = 1). In five of six cases, only one surgery had been performed without recurrence after first-line treatment, while in only one case revision surgery had been performed twice. Overall satisfaction with therapy was high, with the grades for contentment with therapy being distributed as follows: 14/46 (30%) grade 1, 29/46 (63%) grade 2, and 3/46 (7%) grade 3.

## Discussion

Since the first description of unicameral bone cysts by Virchow in 1876, different treatments have been proposed and evaluated. However, up to this day, there is no agreement on a successful standard therapy. A wide range of methods is available, from rather minimally invasive techniques such as aspiration and injection of steroids or bone marrow to more radical open techniques. All of these methods share a considerable percentage of recurrence. Our findings suggest that overall long-term results of surgical therapy are satisfactory. This perception is shared by the few studies providing long-term results of UBC treatment [37,38]. These results demonstrate that regardless of the four treatment strategies used in this study, therapeutic success will likely arise. However, as our data demonstrate, a considerable rate of recurrence can be expected during first, second and even third-line therapy. It is therefore important to scientifically evaluate whether certain methods present a significant reduction in recurrence in the future.

Limitations: Our study population is comparable to other studies regarding age, gender ratio and incidence of pathologic fractures [18,39-41]. Like all retrospective studies, the study has certain limitations, e.g. regarding patient selection. However, unlike in most other retrospective studies, we were able to include a total of 46 out of 62 patients in which both radiographic material and long-term clinical information was available for analysis and was systematically revised. The outcome was objectified by quantitative radiological measurements. Histopathological diagnosis was obtained in all patients; therefore, although presenting a rather average amount of cases, results are not falsified by inclusion of tumours of other origin. Due to the small numbers of patients being treated with screws, methylprednisone and curettage without bone grafting, a comparison of the different therapies applied is not feasible. However, the results of these therapies were included to be compared to other studies and because many of the more recent studies on UBC therapy have provided information on a juxtaposition of these techniques.

In our study, pathological fractures were significantly more often located in the humerus than in the femur, comparable to previous studies [26]. It is believed that due to the absence of a significant weight load, which usually leads to significant pain, UBC in the humerus remain undetected longer than in the femur. Thus, UBC in the humerus often grow until they reveal themselves through a pathological fracture usually arising in the context of a minimal trauma.

We were able to detect significant risk factors for recurrence already depicted in important previous studies, such as male sex, young age [31,36] and active cysts [15,18]. To our knowledge, this is the first study to report that recurrence after more than 30 weeks past surgery is correlated with lower re-recurrence rates in revision surgery. It can only be postulated that less expansive properties of these "late-onset" recurrent cysts may be responsible for this effect.

Almost all of the patients in our collective were treated with curettage and bone graft, either for primary therapy, or for revision surgery. This procedure showed a recurrence rate of 37% (13/35). Previous studies have shown recurrence rates of 20 to 40% for this technique [18,31,35]. Hunt et al. experienced healing rates of 75% for the first procedure and 95% for the second procedure of percuteaneous drainage, curettage and a combination of auto- and allograft [42]. In this study, there was no significant difference in recurrence between the groups treated with autologous graft or allograft. Use of autologous and allogenic bone graft has been discussed most controversially. Obtaining autograft from the tibia or the iliac crest is likely to lead to significant morbidity. In some institutions, this explant morbidity has induced certain reluctance towards autografts, particularly in children. On the other hand, allografts are estimated to exhibit reduced osteogenic properties when compared to autografts [35]. Some authors therefore propagate superiority of autologous bone [43,44]. Also, even in acellular bone, the potential risk of infection allocated with allograft transplantation has generated some restraints to its use. In our opinion, when discussing autografts and allografts, it is therefore important to consider that in our study, bone healing could be effectively attained with allografts.

Because of the disadvantages of both allo-and autografts, bone substitutes have become widely used in adult orthopedics and traumatology treatment in the last decades [45,46]. Yet there is little evidence of their efficacy and studies comparing bone substitutes to bone graft or spontaneous bone healing are missing. A recent study reported healing rates of 66% for curettage and calcium sulfate bone substitute grafting and 91% for a combination of curettage, ethanol cauterization, cyst membrane disruption and combined calcium sulfate bone substitute grafting [47]. A healing rate of over 90% in a minimally-invasive approach of curettage and calcium sulfate pellet grafting has also been reported by Dormans et al. [28]. However, these are the first studies to report on bone substitutes in UBC therapy and further studies are required to confirm the promising results. An important question is whether the use of any of the materials specified above is essential for bone healing in these lesions. Hirn et al. reported good results of curettage without bone grafting in benign tumours of the distal femur and the proximal tibia, with a recurrence rate of 11% in mostly giant cell tumours, chondroblastoma and aneurysmal bone cysts [48]. It is unclear if these results can be extrapolated to unicameral bone cysts, but the study can be regarded as evidence for the high self-healing potential of long bones. In our study, only four patients were treated with curettage without bone graft transplantation. A recent study reported healing rates of 70% percent for this technique [49], which applies to our findings concerning curettage combined with bone grafting. In our study however, no recurrence was observed in the group who had been treated with curettage alone. This may be due to a more aggressive curettage than in the cases treated with a combine bone grafting. Aggressive debridement has been shown to significantly reduce the percentage of recurrent cysts in previous studies [50,51]. Localization of the cysts did not seem to account for the results, as the cysts were localized in four different bones. However, due to the small number of patients treated with curettage alone in our collective, the comparison to those treated with bone graft is not legitimate.

In the 1980s and early 1990s, only a small number of patients were treated by corticoid injections in our institution. Recurrence in our study was 66% for this treatment. However, corticoid injections have been reported to show good results by numerous groups [36,52]. Scaglietti, being the pioneer of this treatment, reported healing rates of 70% to 90% [30,34]. More recent studies have reported healing rates from 33% to 41% [47,49]. However, apart from the minimal trauma resulting from the injection technique, considerable recurrence rates and the need for multiple injections have been reported [31,53]. A study comparing injections of bone marrow in 14 and cortisone in 65 patients reported a need for multiple injections in 57% and 49%, respectively [36]. Postoperative fracture rates also vary importantly among the studies, ranging from 2% to over 30% [30,34,54]. These disadvantages are clearly modifying the benefits of this minimal invasive technique.

In the recent past, cannulated screws have established themselves among the therapies for UBC, promising continuous decompression of the cystic cavity [21,22]. Healing rates between 67 and 92% have been reported [27,47,55]. However, other studies suggest that treatment failures and a significant amount of persistent defects after healing are often observed [56]. A combination of cannulated screws with curettage and calcium sulfate grafting was reported to show recurrence rates of less than 10% [47]. In our study, recurrence rate for decompression with cannulated screws was 50%. Again, due to the small number of patients treated with corticoids or cannulated screws in this study, no comparison can be made with the other surgical therapies in this study. However, we chose to include these cases to provide information on long-term clinical results for all approaches. The coexistence of different surgical approaches is experienced both in clinical practice and the literature. In different institutions, the frequency of the therapies applied can significantly vary, according to personal experience and beliefs. Interestingly, out of the six patients with abiding symptoms in 2007, six had received curettage and bone grafting. Among these, five had been successfully treated after only one surgical intervention. This suggests that short- and mid-term success is not to be equalized with a guarantee on patients free of complaints in a long-term consideration.

## Conclusions

We were able to provide long-term clinical outcome for surgical treatment of unicameral bone cysts. Our results suggest satisfactory overall long-term outcome, but short-and mid-term observation showed a considerable rate of recurrence for all strategies applied. To our knowledge, this is the first study to report that patients experiencing recurrence later than 30 months after surgery have a better prognosis regarding revision surgery. Considering the almost assured long-term success of UBC therapy, invasive strategies have to be carried out as cautious as possible.

## Competing interests

The authors declare that they have no competing interests.

## Authors' contributions

SH, FZ and BL conceived of the study. SH drafted the manuscript. FE provided patients information and collected the data required. FE and SH performed the radiological analysis and the statistical analysis. BM, TD, TG and BL participated in study design and coordination and helped to draft the manuscript. All authors read and approved the final manuscript.

## Pre-publication history

The pre-publication history for this paper can be accessed here:

http://www.biomedcentral.com/1471-2474/12/281/prepub
